# Three-level containment model of hospitalized adolescents with borderline pathology: a holistic therapeutic perspective

**DOI:** 10.3389/fpsyt.2023.1110788

**Published:** 2023-08-07

**Authors:** Marion Robin, Laura Bellone, Jean Belbèze, Koucha Kazemian, Rahmeth Radjack, Maurice Corcos

**Affiliations:** ^1^Department of Adolescent and Young Adult Psychiatry, Institut Mutualiste Montsouris, Paris, France; ^2^Paris-Saclay University, UVSQ, CESP, INSERM U1178, Team PsyDev, Villejuif, France; ^3^Paris Cité University, Paris, France; ^4^Maison de Solenn, Department of Adolescent Psychiatry, Cochin Hospital, AP-HP, Paris University, Paris, France

**Keywords:** adolescents, hospitalization, holistic model, thee-level containing, borderline personality

## Abstract

Borderline personality disorders account for 50% of adolescent hospitalization cases in psychiatry. The severity and psychopathological complexity of these symptoms indicate the need for inclusive models of understanding. Adopting a holistic approach allows for the consideration of not only the patient's environment, but also their position within that environment and their life history. In this article, a model based on the concept of therapeutic containment at three levels is presented. Global containment refers to the mindset and organization of the institution that provides care, which is itself a part of society at a specific time. Local containment focuses on understanding and therapeutic interventions within the immediate social environment of the individual. Lastly, individual containment encompasses the development of independent processes during the course of care. These three levels are integrated in the hospital treatment of borderline personality disorders, forming a trans-theoretical approach.

## Introduction

Adolescent psychiatry is characterized by its richness, variability, and the complexity of its symptoms. Models of understanding cannot afford to be exclusive due to the multifaceted nature of the adolescent experience. The relational function of symptoms prevents us from perceiving the adolescent as an isolated or independent entity. Acts and transitions to action challenge the notion of the adolescent as a purely thinking subject. The development of reflexive faculties at this age contradicts the idea of an all-acting subject. Frequent somatizations remind us that the mind cannot be detached from its bodily anchorage. The need for medication highlights the importance of the relational aspect. Furthermore, the acuteness of family conflicts and traumas underscores the group and systemic dimension of symptoms. The practice of adolescent psychiatry goes beyond integration or holism, as it encompasses the environment and the overall journey of the individual, utilizing various theoretical tools.

Borderline Personality Disorder (BPD) is closely intertwined with the environment. Its etiology is complex and influenced by multiple factors. The biopsychosocial model has gained consensus, emphasizing the continuous interaction between the individual and the environment in the development of BPD (1). Epigenetics research supports this view, particularly stress-diathesis models that consider the interplay of genetic vulnerability and the effects of life experiences (2, 3). In addition to genetic, neurobiological, and temperamental factors (4), the occurrence of adverse events is well-documented in borderline pathology (5–7). From its inception in the late nineteenth century to current classifications, BPD has been characterized by its intricate relationship with life events, under the concept of psychogenicity. The clinical description of borderline pathology establishes a strong connection between patients' symptoms, their environment, and the relational events that shape their turbulent journey. Borderline individuals are in a constant state of interaction with their environment, which significantly influences them. The inherent fragility of the self makes them porous to every element in their environment, almost like mirrors. Additionally, healthy adolescents and patients (adolescents or adults) with BPD share specific characteristics in terms of psychic functioning and dynamic conflict movements, such as issues with autonomy and dependence, increased sensitivity to intrusion and abandonment, cognitive functioning with a Manichean tendency, and self and relationship instability, among others. Adults with BPD seem to extend an unresolved adolescent process indefinitely (8). Thus, there exists a continuum between adolescence, borderline psychopathology, and society. Our clinical work aims to establish a continuous process of differentiation and gradation, taking into account these various aspects in a holistic manner.

BPD is a complex psychiatric condition characterized by a wide range of symptoms, all revolving around the theme of instability and oscillation. This includes oscillation between anxieties of intrusion and abandonment, instability of the self and relationships, impulsivity, feelings of rage and emptiness, as well as anxious and mood symptoms. Clinical presentations of BPD vary, but are often marked by acute episodes that may involve anxiety, self-mutilation, suicidal attempts, dissociative episodes, and more. These moments of clinical severity, with their potential prognostic implications, are the primary reasons for hospitalization in psychiatry, accounting for 30 to 50% of hospitalized adolescents (9). The coexistence of these noisy but episodic clinical moments with underlying and enduring psychopathological processes necessitates a two-level analysis: nosographic and psychopathological, or categorical and dimensional. In this regard, it remains useful to distinguish between BPD as defined by DSM criteria (characterized by overt symptoms such as self-destructive behaviors, uncontrollable anger, impulsivity, etc.) and borderline functioning in the psychoanalytic psychopathological sense, encompassing a borderline state/personality organization (involving defense mechanisms such as splitting and projective identification, anaclitic dependency relationships, ego porosity with intra-psychic conflicts between intrusion and abandonment, etc.) (10). It is possible to experience one without the other, and often, but not always, adolescence reveals a borderline crisis with symptoms that have been evolving since childhood. Thus, we refer to an “adolescent borderline crisis,” which may complicate an earlier borderline functioning (11), reserving the term “BPD” for situations that confirm a categorically observable functioning persisting into adulthood. The therapeutic perspectives outlined in this article apply to the entire field of borderline pathology.

These moments of clinical and prognostic severity, where the vital prognosis is at stake, often lead to the hospitalization of patients with borderline symptoms. Self-destruction, which is a challenging symptomatic dimension to address in psychiatric consultation or psychotherapy (12), frequently necessitates hospitalization. For adolescents with borderline symptoms, the duration of hospital care can be divided into three distinct periods, with some overlap in timeframes, each lasting approximately one week.

The first period of hospitalization focuses on global containment. This involves providing reassurance to the adolescent and limiting their internal or externalized agitation, anxieties, and resulting disorganization. Achieving global containment in the hospital requires various measures, including architectural considerations (department structure), organizational aspects (care planning), and institutional factors (team mindset and ensuring team safety, based on the clinical assessment of the patient).

- Once global containment has been established, the second phase of care involves local containment. During this stage, the focus is on the adolescent and their immediate relational environment, with the goal of understanding the factors contributing to the deterioration of their psychological state. This includes identifying the psychological events that have led to the current situation, which may have been challenging and unchanging for weeks, months, or even years. Additionally, the general context of the adolescent and their typical level of functioning is evaluated. This phase of the work entails exploring emotions and representations, analyzing and mentalizing experiences, and fostering individual and collective sharing.

- Finally, the last phase of care, individual containment, combines a gradual increase in patient autonomy to prepare for discharge and the development of a more active role in their own care. Therefore, the objective is to facilitate the patient's departure from the hospital by finding a satisfactory compromise that considers the needs and desires of all parties involved, including caregivers and parents. This is the time when the young person reconnects with friends and teachers, organizes their return to school, and coordinates their ongoing psychiatric care. The separation from the care team at the time of discharge is smoother when the focus of the therapeutic exchanges has shifted from symptoms to the sharing of positive and constructive experiences.

These three periods of care correspond to three distinct levels of therapeutic intervention, drawing from various theoretical perspectives that complement each other. They are organized according to the global, local, and individual levels, ranging from the broader containment to more focused interventions. Given that psychotherapy models may not be accessible in all geographical areas and that patients with severe disorders may have difficulty engaging in regular psychotherapy, our model aims to integrate different therapeutic approaches, which can be particularly beneficial for these challenging cases. These approaches serve as the foundation for achieving the three therapeutic objectives in BPD that align with the three levels of care organization: 1. stopping self-destruction, 2. identifying and addressing the factors contributing to the crisis, and 3. recognizing and connecting emotions to their triggering events to regulate them through bodily, psychological, and relational processes.

## Global therapeutic containment

To prevent self-destruction and facilitate the patient's personal growth and interpersonal connections, a crucial resource is the supportive institution. In order to fulfill this role effectively, the institution must possess specific characteristics, as outlined by the theoretical framework of institutional psychotherapy.

### A. Institutional care mindset

Over the last 70 years, theorists of psychiatric care have discovered, with institutional psychotherapy, the extent to which the state of mind and the application of an active and organized collective life is a fundamental institutional prerequisite for receiving and caring for a patient (13). Although these practices were thought of with adult patients suffering from psychotic disorders in mind, certain principles are completely relevant in adolescent psychiatry, particularly with patients with borderline symptoms (14).

In the hospital, death distress is the daily life of the psychiatrist who treats borderline pathology. These workers are the ones to whom adolescents convey their suicidal behaviors, their negative feelings toward their entourage, and toward the caregivers who are in contact with them. The psychological work often revolves around understanding the stakes of the individual's situation and their family history concerning death. It always deals with a group, in this case a family, and the way in which death distress is dealt with within this group. A quarter of adolescents (24.6%) with DSM-5 criteria of BPD have a parent who has attempted suicide (15). The psychiatric work is also linked to the analysis of the history, the narration, and the re-appropriation of the individual and collective parts of the fear of separation and death. The group device constitutes a therapeutic space in the spirit of “transitionality”, as developed by Winnicott. It is a space in which patients who are in a state of crisis are able to express the most archaic and stormy strata of their thoughts and it can open or reopen “the way to a sufficiently existential foundation” (16), especially when the singular therapeutic relation between the patient and his psychiatrist remains unsuccessful for a real “psychic accommodation” (17). However, before all this work intending to give the patient's story a narrative can begin, first, one must consider protecting the patient against acts of self-destruction, and thus ensuring their survival. At the very beginning of the care lies the necessity to stop the escalation of suicidal behaviors and the escalation of danger and fear.

The psychiatrist overseeing the patient's treatment must effectively manage the adolescent's behavior to ensure their safety and wellbeing. Initially, measures such as restricting the patient's freedom of movement, removing dangerous objects, and ensuring a secure environment are necessary steps in providing care. However, it is crucial to recognize the limitations of these restrictive measures and to determine when it is appropriate to gradually reduce them, allowing the patient to regain their autonomy and sense of responsibility. As the patient's clinical condition improves, typically within a few hours or days, the care team can work toward preserving the patient's privacy, freedom of movement, and encouraging their active participation in decision-making. It is important to strike a balance between maintaining safety and avoiding excessive control, as an excessive focus on absolute safety and eliminating all risks can actually be counterproductive and reinforce suicidal symptoms. By excessively exerting control, caregivers inadvertently reinforce the patient's pre-existing feelings of powerlessness and contribute to a sense of intrusion that further intensifies their fears. Therefore, it is essential for caregivers, in addition to the psychiatrist, to navigate the concept of risk and help adolescents gradually overcome their risk-avoidant thinking (often characterized by all-or-nothing reasoning) that is inherent in borderline psychopathology.

### B. Organization of institutional care

In the tradition of F. Tosquelles and H. Simon, the institutional organization of care aims to address three threats to patients' wellbeing: inaction, an unfavorable hospital atmosphere, and the prejudice of patient irresponsibility (13). Hospitalized patients engage in various mediation activities such as visual arts, rock climbing, relaxation exercises, meditation, singing, and circus arts. These activities provide patients with an active role within a group, fostering interdependent relationships and collective creativity. The focus shifts from the patient's illness to their strengths, aligning with Jean-Baptiste Pussin's approach in the eighteenth century. These workshops offer patients an opportunity to explore aspects of their personality that provide personal pleasure, fulfillment, and unique experiences (18). During these experiences, patients are both differentiated from others and connected to them, facing the complex challenge of maintaining individuality while remaining connected. The mediated group setting facilitates symbolic expression without the therapeutic relationship becoming overly threatening. The use of artistic media allows patients to “play and give form” to their relationship with the clinician (19). As caregivers also participate in workshops outside their expertise, these mediation sessions promote a more horizontal caregiver-patient relationship. It encourages a co-construction of care rather than a hierarchical medical approach that can be perceived as powerful and intimidating. The attachment insecurity often observed in patients with borderline pathology complicates the provision of help, as they may simultaneously seek and reject it. Moreover, this defense mechanism evolves in response to the actions and words of others, creating an idealized or demonized image of oneself and others, further amplifying the perceived power of medicine and caregivers (the power to heal magically or harm diabolically). Therapeutic efforts are more effective when adapted to include the patient as an active participant in their healing process, thereby alleviating the overwhelming sense of powerlessness experienced by patients who have endured numerous traumatic experiences.

A weekly multidisciplinary synthesis meeting is organized to gather insights from various caregivers, serving three fundamental functions. Firstly, it facilitates the exchange of information about the patient that goes beyond their symptoms and daily care, including details the clinical significance of which may not yet be fully understood. Secondly, it provides an opportunity for caregivers to express their emotions and delve deeper into the emotions evoked and projected by the patient. Lastly, it serves as a collective platform for developing the care project. The fragmented nature of borderline identity presents challenges in providing care, as different fragments of the patient's personality are projected onto different caregivers throughout the course of hospitalization, referred to as the “transferential constellation” (20–22). Establishing trust in care is difficult for caregivers, and for the patient, investing in a relationship with a care team is less intimidating than investing in a relationship with a single individual. Similarly, caregivers quickly encounter their own powerlessness in providing “good care,” and teamwork serves as a support system, enabling shared experiences and the ability to navigate this feeling without jeopardizing the therapeutic relationship. By identifying the nature of exchanges, emotions, and psychological conflicts that unfold during the synthesis meeting, the care project begins to take shape. The collective unconscious plays a role, and the sharing of representations fosters the emergence of new ideas. Collective intelligence promotes the appearance of these emerging processes, which unfold in unpredictable ways, even though the therapeutic setting influences their probability of occurrence (13). These processes confirm that the representations generated by the group are greater than the sum of their parts. To facilitate the emergence of the patient's true self from their symptoms, the fragmented elements must first be gathered, re-associated, and reorganized. Cleavages and projections are the challenges that compromise coherence and continuity, and caregivers work to address them. Through the synthesis of the elements of the transferential constellation, they aim to give it shape and assist the patient in constructing a coherent sense of self (10). This work creates a space for the patient to acquire or reclaim the subjective position that may have been lost beneath the symptoms (13).

### C. Recognizing the societal factors influencing self-destructive behaviors

We have observed that clinicians have long established connections between borderline symptoms and the environment. However, the significant increase in suicidal behavior among adolescents since 2020 has driven research to identify psychopathological correlations between societal factors and the psychological wellbeing of adolescents. While it is not to say that society creates and solely explains psychological disorders, it can be considered that society influences the expression, development, resolution, and acceptability of these disorders to varying degrees. Psychological disorders can manifest differently depending on the societal context in which they arise, and they may also occur more frequently if society lacks the protective barriers that typically contain fear, anger, and sadness. In this sense, if we conceptualize institutional containment as that of society, the state, a group, or collective organization, it enables proactive intervention in the best interest of individuals. Furthermore, gaining a comprehensive understanding of the relationship between these societal factors and patients' symptoms is a crucial prerequisite for any therapy. On one hand, recognizing these phenomena at the collective level allows for their discussion with the patient and their family, who will experience them in a unique but not isolated manner. On the other hand, this holistic understanding of the environmental effects enables the establishment of prevention policies that have individual-level consequences and, if effective, reduce the need for individualized care. Therefore, preventing suicidal behaviors in adolescents, including those related to borderline crises, requires an understanding of these mechanisms that encompass both the adolescent themselves and the institutions responsible for their care. It is also important to avoid interpreting all problematic phenomena in adolescents solely from a medical perspective, as this would underestimate other crucial causal factors that are societal rather than medical in nature. Examples of such factors include social inequalities, territorial policies, child protection, and the impact of screen usage on cognitive functions. Hence, it is the responsibility of caregivers to bear witness to the various factors contributing to their patients' disorders, with the goal of addressing them collectively at a societal level in parallel with individual treatment.

Among the various societal factors, the COVID-19 pandemic has been identified as an immediate explanation for the significant increase in suicidal behaviors among adolescents. The restrictions imposed during the pandemic had a profound impact on the global population, particularly hindering adolescents who have a strong need for exploration beyond the family environment. This period of withdrawal and fear of others not only led to an increase in suicidal issues but also manifested in symptoms related to fear and control, such as phobias, anorexia, and somatization. It is important to note that even before the pandemic, the isolation of young people was recognized as a societal problem (23). However, the quality of family support and social integration plays a significant role as a prognostic factor in suicidal problems. The fragility of such support, which varies from country to country, contributes to the sense of isolation in young people who rely more on virtual relationships rather than in-person connections. Since 2020, alongside the reinforcement of social distancing measures, adolescents have experienced a notable increase in symptoms of “derealization” and dissociation, which are characteristic symptoms of borderline personality disorder (24). These symptoms reflect an overwhelming feeling of detachment from oneself and reality, where individuals no longer feel present in their own bodies. While these symptoms are severe, they can be alleviated through the use of words, self-care, in-person relationships, and shared activities. Many solutions exist outside the realm of medicine to address these issues. In some countries, the suffering experienced by adolescents and the timing of their symptoms, when correlated with the school calendar, reveal a significant amount of school-related pressure. This pressure, combined with frequent cases of bullying in the school environment, contributes to the adolescents' sense of inadequacy. It highlights the challenges they face in hierarchical and competitive systems that prioritize conformity over personal development and lack sufficient opportunities for encounters and cooperation. Additionally, instances of neglect and abuse, both within and outside the family, exist and are often inadequately addressed in terms of prevention and care. These situations are now highly medicalized in several countries, and the lack of medical resources to address the increasing rates of suicidal acts further hinders caregivers from fulfilling their role as a last line of defense against suicide. Providing a welcoming, reassuring, and present presence over time and ensuring continuity of support are crucial aspects of this role, but are often impeded by resource limitations.

At a family level, the deployment of parenthood is affected by professional and personal stress, which is further exacerbated by the lack of adequate social and community support. This hinders the collective effort in raising a child, leaving isolated and inadequately supported adults to shoulder the responsibility alone. However, isn't it said that “it takes a village to raise a child”? The pervasive experience of death and distress during the pandemic has underscored the crucial importance of psychological resilience and the need for a collective social project that goes beyond mere survival. In times of adversity, and in life in general, the psychological suffering arising from a situation is often more closely tied to the absence of meaning (shared values and objectives) rather than the situation itself. Without a sense of purpose, any stress-inducing reality can become traumatic. Young people are acutely aware of the stakes involved in climate change and the preservation of life, and their sense of isolation grows when adults fail to protect the planet and the natural world. This sense of immobilization and denial creates a feeling of dissociation between the recognition of a major threat and the failure of adults to safeguard the future. Furthermore, the actions of young people are sometimes criticized and belittled. However, engaging in civic activism is one of the most effective ways to foster social integration and combat anxiety and powerlessness. For society, youth represents a wealth of fresh perspectives, an impetus for action and creativity, and the primary transformative potential, provided they are given the necessary opportunities to fulfill their role.

Outlined here are the societal issues that can influence the psychopathology of destructive behaviors, prompting us to consider collective action for the prevention of mental health problems. Such efforts would significantly support institutions in maintaining their therapeutic role in alleviating patients' suffering. However, it is crucial not to oversimplify borderline pathology as a purely psychic or purely social phenomenon, but rather to recognize its multifaceted nature. Examining the immediate relational environment of these individuals helps us comprehend how the interaction between the individual and society unfolds, with the ultimate goal of informing therapeutic interventions.

## Local therapeutic containment

### A. The assessment of the relational environment

Given the chaotic environment typically experienced by adolescents with borderline symptoms, the therapeutic management of these patients requires a comprehensive assessment of their relational history. It is crucial to address environmental challenges in addition to therapeutic interventions, often involving medical and child protection measures, as solely focusing on therapy may prove insufficient or ineffective. During hospital treatment, significant time is dedicated to conducting medical interviews with both parents present. These interviews serve to explore the family and patient's history, including psychiatric, medical, and legal aspects, as well as assess the difficulties and level of parental distress. Therapists actively encourage the psychological and relational engagement already initiated by the psychiatric crisis and hospitalization. In this way, they initiate treatment of borderline symptoms by incorporating the patient's environment and evaluate the family's capacity to adapt to change, engage in self-reflection and questioning, and collaborate with professionals, all of which are important prognostic factors for adolescent borderline pathology.

Early adversity in borderline pathology has a significant impact on neural and cognitive functioning associated with the stress axis through epigenetic mechanisms (25–27). It also disrupts attachment patterns, which in turn contribute to the development and perpetuation of borderline symptoms (2, 28). Research has shown that severe childhood maltreatment is prevalent among adult patients with BPD, and similar findings have been observed in adolescents (29). This maltreatment includes traumatic life events, abuse, and dysfunctional parenting. Examples of traumatic events include early separations, childhood loss, bereavement, and severe threats of loss such as parental suicide attempts. Maltreatment often involves emotional abuse, including child denigration, as well as sexual abuse (found in 30–90% of cases and 28.6% of borderline adolescents) (15, 30), and neglect. A significant number of hospitalized young people face challenges in their immediate environment, where their basic needs beyond material necessities are often overlooked. These needs include shared time, attention, emotional care, consistent rules, and support.

Parenting dysfunctions are characterized by controlling behaviors and a decrease in the level of emotional care. Problematic interactions, such as emotional invalidation, suicidal threats in front of the child, repeated conflicts revolving around the child, and loyalty issues between parents, are also common (1, 15). These adversity factors play various roles in the psychogenesis of borderline pathology, with their accumulation associated with the severity and early onset of symptoms (30). Furthermore, their variable combinations contribute to distinct symptom patterns and clinical presentations (15). The cumulative effects of these factors contribute to the complex trauma experienced by adolescents with borderline pathology. Such trauma involves early and prolonged experiences that often involve attachment figures. Like other forms of complex trauma, the consequences include not only dissociative trauma symptoms but also profound identity shifts and attachment disorders (31).

Hospitalization frequently provides an opportunity to identify or observe evidence of ongoing or past abuse. Caregivers play a fundamental role in establishing an educational framework during hospitalization, while recognizing the importance of involving educational services regularly used during treatment. The aim is to create conditions that promote a calmer and more supportive parental environment for the adolescent. This approach includes supporting both the parents and the adolescent in addressing their limitations, while respecting each party's subjectivity and needs. In most clinical situations, caregivers emphasize the importance of meeting the adolescent's emotional needs in an age-appropriate manner. This includes providing a reliable and predictable environment, supporting autonomy in a gradual manner, setting parental expectations based on the child's abilities, and ensuring shared time, attention, emotional care, support, and a structured framework with clear rules. Observing these parental difficulties often prompts clinicians to establish close collaborations with child protection services, which may vary in difficulty depending on the country. Such collaborations are necessary to break the cycle of abuse or neglect. In addition to the responsibilities of child protection services, psychiatric professionals systematically consider and address these relational factors to establish an environment that promotes the adolescent's recovery.

### B. Considering the systemic dimension of symptoms

The presence of adversity should be considered at both the individual and family levels (32). Exploring the family history in the presence of the adolescent can be therapeutic from the beginning. Admission to the hospital often unveils a suicide attempt by a parent or other family member during the medical interview. Conflicts between the adolescent and their parents are frequently linked to relationships between the parents and members of their family of origin, such as siblings or parents. Parents often describe complex relational issues with siblings or parents that have led to major conflicts and unresolved life-and-death matters. In these interviews, the adolescent realizes with relief that they are not the root cause of the problem as they had believed, but rather that their symptoms represent a variation of it. Traumatic relational elements leave their mark differently across generations and time, and the adolescent's symptoms provide a gateway to accessing these hidden crypts and secrets that are passed down (33, 34). The adolescent, who carries the symptoms, becomes the person who enables the family to access care and is valued by the care team for this role.

Working with family conflicts is akin to working with trauma. Patients and parents recount life situations that have remained unresolved, painful, and immobilizing. During these narratives, emotions emerge and become associated with the words and shared representations. Expressing emotions, connecting them to representations, and sharing them with others are the essential steps to move beyond a traumatic state. Conducting three to five one-hour interviews with the patient and their parents together often results in a significant change in family dynamics. The crisis situation, with its deep family imbalance, simultaneously encourages mobilization and resilience. Although hospitalization may be a moment of suffering and imbalance within the family unit, it also opens up possibilities for a new and better-adjusted equilibrium.

It is widely accepted that the therapeutic management of BPD in adolescence requires significant involvement of the family (35, 36). The exploration of family dynamics begins with the adolescent's symptoms. The family is encouraged to establish hypotheses about factors that are somehow related to the symptoms or the adolescent's overall distress. Starting from the symptoms, an associative thread is woven, leading back to the history, the “pre-history” of the adolescent, and various family members, particularly addressing the knots, conflicts, and impasses within this collective history. The suicide attempt, a central symptom of borderline pathology, represents not only a refusal to live but also a message directed at emotional support, indicating a resignation to jointly find a solution to pre-existing distress. It signifies the loss of hope in the relationship and a desire to detach oneself from others during this overwhelming distress to regain some control that has been completely lost and to preempt an unbearable fear of abandonment. The suicide attempt also reflects an anger that cannot be directed toward someone who would not hear or support it. Borderline anger represents an inward turn of aggressiveness, and the role of the psychiatrist is to explore with the patient and their parents which feelings could not be identified and expressed to their family and friends.

In some cases, a suicide attempt can also reflect an intense feeling of self-disappointment, often associated with an excessively demanding ego-ideal. The adolescent may feel that they are not living up to the expectations placed upon them by their family, such as achieving academic success. This self-disappointment is a sign of introjection, where the adolescent internalizes the demands of society, often conveyed through their parents. Adolescent psychopathology shows that what we commonly refer to as “school failure” becomes a matter of life or death when it is accompanied by a sense of rejection. Unlike a healthy individual who may respond to the failure of their ideal with modesty, patients with borderline pathology experience it with depression. The externalized ego-ideal is gratified through action (37). The adolescent perceives that their failures could bring shame to their family and friends, or that society excludes them. Consequently, they contemplate death as a means of protecting themselves. What the adolescent truly seeks is not merely a diploma, but rather the unconditional love and acceptance of their parents, as well as a sense of belonging in society. They long for the certainty of being loved regardless of their abilities and for gaining collective esteem regardless of their social status. They shoulder the immense weight associated with these expectations, to the extent that it becomes a matter of life or death, particularly when there are no supportive structures in place. This leaves the patient trapped in their anxieties without the possibility of overcoming the issue of “success” and developing self-actualization (38). Adolescents who struggle academically and contemplate death are expressing that their need for emotional safety outweighs their need for scholarly success.

Family relationship work is highly beneficial in the care of adolescents, but it often evokes feelings of guilt that need to be identified and addressed. This guilt stems from the awareness of the affective power of interpersonal relationships on oneself and others, which patients may alternately minimize or exaggerate through a process of splitting. One way to alleviate overwhelming guilt is for parents and doctors to sometimes seek genetic or biological explanations for the pervasive nature of the disorder. However, parents are relieved when they overcome the difficulties associated with guilt and are able to identify the unresolved wounds from their own history that resonate with and contribute to their adolescent child's difficulties. They appreciate the opportunity to comprehend the emotional and transgenerational issues that have hindered their relationship with their child, as it empowers them to influence the relationship at both an individual and family level. Medical interviews with parents involve psychoeducational work on the diagnosis, characteristics, and evolution of the disorder, which also helps alleviate parental guilt (39). Multifamily groups and individual therapy can be utilized to teach parents how to overcome an invalidating parenting style (40). Additionally, siblings may be included in consultations as well, as borderline symptoms affect the entire family, and siblings often remain silent to avoid exacerbating the problem.

Moreover, the process of finding meaning and understanding the functions of symptoms is not contradictory to the search for a biological or genetic cause, or the implementation of solutions that may include medication. It involves attributing complexity to situations and moving beyond binary reasoning—cause and effect—to embrace circular and systemic reasoning. After a suicide attempt, patients often identify one or two causes for their actions, while their parents identify two or three causes, which may not necessarily align. However, after a few weeks of hospitalization, caregivers can deconstruct a dozen causes or, rather, conditions that contribute to the emergence of suicidal thoughts or behaviors (38). No single factor alone determines the outcome, but the convergence of these factors creates the possibility of a suicidal event or symptom. Once these conditions have been identified, caregivers work collaboratively with patients and families to develop solutions, whether they are psychological, physical, or involve adjustments to the adolescent's daily life (e.g., treatment approaches, living arrangements, school accommodations, etc.). However, caregivers often encounter resistance if they propose quick-fix solutions. The concern or distress may shift from one issue to another, and simple adjustments may not be suitable until the underlying relational needs are understood and the solutions are co-constructed. The stability of the family system can challenge the therapeutic alliance, and this reality is openly addressed with families as a process linked to the complexities of therapeutic responses. The conditions that contribute to the emergence of symptoms reflect a range of unmet needs that could not be expressed in any other way, but are now being explored to address them proactively before they manifest as symptoms. The adolescent assumes the role of the person who enables the identification of these dysfunctions and is valued for fulfilling this role. The more the family can establish themselves in this process, shifting their focus from the adolescent's symptoms and reorganizing their functioning, the better the prognosis for the patient. This family work occurs on two levels: the internal reality level, which involves addressing individual and group unconscious conflicts, and the external reality level, which involves concrete interventions to adapt the educational framework to meet the adolescent's daily needs.

Systemic family therapies are commonly prescribed for the majority of families following hospitalization, when there is a capacity for psychological mobilization and the therapeutic work is deemed significant. However, not all families of adolescents with borderline pathology possess the minimum degree of cohesion required for this type of therapy. Additionally, a highly projective group dynamic can render this work unfeasible. Other specific therapeutic interventions have been developed to support parents or the family, focusing on particular areas such as psychoeducation based on Dialectical Behavioral Therapy, mentalization processing, or trauma-focused approaches (39, 41–46). Family interviews conducted during hospitalization can already address various dimensions, including reducing educational inconsistencies, reviewing parental and adolescent expectations, identifying and interrupting role-reversal dynamics, reestablishing protective roles, decreasing levels of control and criticism, promoting emotional validation of the adolescent by their parents, identifying issues related to relational distance (separation or intrusion), recognizing paradoxical communications, addressing conflicts, and highlighting transgenerational repetitions.

### C. Psychoeducation

At this stage, the issue of diagnosis arises, either at the request of the parents or with the aim of clarification, therapy, and psychoeducation. While diagnosis is not a routine practice in adolescents, mainly due to the risk of pathologizing identity formation during this critical developmental period, it can still be valuable in many cases. Its utility should be evaluated on a case-by-case basis. Diagnosis can guide the treatment team during and after hospitalization, such as determining the need for a day hospital program or requesting disability recognition. However, its primary benefit lies in helping the patient and their parents organize their responses to the symptoms with a prognostic perspective. Experiencing a significant stressor like a suicide attempt or self-injury in a child necessitates that parents project themselves into the future and avoid succumbing to despair. However, the diagnosis of BPD inherently implies a prognosis extending into adulthood, which may not align with the duration of the most overt and disruptive BPD symptoms. Therefore, when diagnosing BPD in adolescence, it is crucial to clarify the precise nature of the observed symptoms, including emotional, relational, behavioral, and identity-related instability. Additionally, underlying psychopathological elements should be addressed, such as increased dependence, excessive reliance on relationships at the expense of one's own needs, and diminished self-esteem and confidence in one's abilities. It is important to explain to parents the diagnostic challenges in balancing the concept of a personality disorder with the scientific predictive validity of the disorder. Statistically, approximately three-quarters of individuals with BPD experience significant symptom remission within 2 years. This has been supported by studies conducted by Bernstein et al. (47), Levy et al. (48), and Miller et al. (49). Furthermore, the proximity of certain features of adolescent crisis to BPD symptoms may have led to an overestimation of the disorder's prevalence, which is actually estimated to be around 3% instead of the previously suggested range of 6% to 18% (47, 50–52). Borderline behavioral symptoms can overlap with symptoms present in other disorders, such as eating disorders, substance use disorders, or adolescent depression. The expression of these symptoms may be more prominent at the physical level during this developmental stage, including self-injury and impulsive suicide attempts (47, 53). The co-occurrence of these symptoms may have contributed to the overestimation of BPD. In such cases, the duration of borderline-type symptoms may be shorter than that of the co-occurring disorder, leading the concept of a personality disorder to hold less relevance for the patient, parents, and healthcare professionals.

For these reasons, it is more appropriate and cautious to use the term “borderline crisis in adolescence” (11, 54), which captures the symptomatic behavioral presentation while incorporating the scientific elements of predictive validity for the disorder at this age. This approach allows for differentiation and compatibility between categorical and dimensional perspectives, both of which are necessary for medical evaluation. The term “borderline crisis” describes the presence or absence of symptoms according to international nosology, while “borderline personality functioning” describes the underlying traits that should be targeted in long-term therapeutic interventions. This diagnosis also enables the naming of the clinical state in real-time without having to wait for a year or more (as required by DSM criteria for diagnosing BPD in adolescence), thus facilitating specific and prompt interventions during crises. Furthermore, in certain situations, it is particularly useful to inform parents that their adolescent does not have a psychiatric diagnosis or does not meet the criteria for BPD, especially when the environment's perception of illness undermines the patient's speech, desires, or choices. This requires clinical and therapeutic sensitivity to reassure the family and help them identify precise transgenerational traumas in order to establish more realistic expectations for their child's future. Regardless of the diagnosis, it is crucial to avoid exclusively focusing on the adolescent with BPD (36). They should be seen as part of the solution rather than the problem, and the entire family system needs to modify roles and behaviors to foster cohesion. Psychoeducation can be incorporated alongside symptom defocusing, even after a diagnosis has been made, to ensure the importance of transgenerational work is not overlooked despite the need for psychoeducation.

### D. Restoring a balanced relationship with others

Attachment theories, such as the strange situation paradigm, have taught us that one-year-old infants respond to brief separations from their primary attachment figure in specific ways (55–57). When an adolescent is hospitalized, they encounter strangers and experience multiple separations from and reunions with their parents. From an attachment perspective, this situation is also relevant. Although the temporality differs, it allows for comparisons, and the precise analysis of the patients' attachment patterns (secure, ambivalent, avoidant, or disorganized) is essential, given that attachment insecurity characterizes 96% of hospitalized adolescents (58). Thus, psychiatric hospitalization at this age becomes both an opportunity to assess attachment security and a therapeutic tool to experiment with separation in a controlled environment, with familiar reference points and with outcomes that differ from experiencing “fear without a solution,” as described by Main and Hesse (58).

Hospitalization also provides an opportunity to mobilize Internal Working Models [IWM, (59, 60)], which are mental representations of the relationships established with attachment figures. These models incorporate beliefs about oneself as worthy of existence and deserving of help, as well as beliefs about others as competent and available when help is needed. Many hospitalized adolescents report a tendency to cope with major life difficulties on their own. In reality, their symptoms maintain a “forced” relational closeness with attachment figures. A central objective of hospitalization is to enable them to integrate the possibility of creating new relational modalities, including seeking support from others. This can be facilitated through interviews, art therapy, medication, shared meals, or informal exchanges with the treatment team. The idea is to help the patient recognize that their current relational patterns, which may have been adaptive in the past, have become dysfunctional. For instance, adolescents with predominantly avoidant attachment are encouraged to reach out to caregivers when they have even the slightest need or difficulty, despite their inclination to minimize and rely on themselves. They are asked to make 5 to 10 relational attempts, such as requesting treatment as needed, asking for time to talk, or discussing their day. These attempts are systematically addressed and analyzed in individual interviews with the doctor and a nurse. Through the gradual re-establishment of secure connections in a safe environment, patients with avoidant attachment can regain or develop the capacity to reflect on their internal states, identify their emotions, and take care of themselves. This process often requires evoking multiple situations in interviews to construct secure representations, as patients initially resist due to their typical avoidant belief of “No one can help you better than yourself” (61).

From a complementary psychoanalytic perspective, listening to and observing hospitalized patients, even at a young age, reveals significant repetitions that unfold in the transference toward the caregivers. These repetitions reflect the patients' past experiences with others. Each patient guides the caregivers toward a specific relational position, which is important to identify, name, and share. This process involves accepting identification with this position and then shifting away from it, opening up creative possibilities for care instead of sterile repetitions of destructive relational patterns. Similar to the role of psychoanalysis in institutional psychotherapy, understanding the psychoanalytic dynamics of the patient's case is not characterized by silence, neutral listening, or verbal interpretation (21, 22). It allows for the elaboration of institutional responses, prescriptions, actions, attitudes, and everything that contributes to the therapeutic context and the patient's care project. However, in adolescence, these relational mechanisms become more complex as transferential processes unfold directly with caregivers and indirectly through parents. These dynamics need to be analyzed, particularly in terms of areas of splitting and projection. These therapeutic procedures resemble Transference Focused Therapy adapted for adolescents (62, 63), which aims to identify internalized dyads of idealized and persecutory object relationships during psychotherapy. These dyads are characterized by oscillating, incompatible, and unconscious self-other representations. The therapist seeks to track their activation and alternation by identifying with whom these movements are “staged” or conveyed (63). These processes need to be analyzed and clarified within the relationships in which they occur (with parents, team members, or peers) before or alongside their direct exploration in the transference itself. Negative therapeutic reactions (64) may manifest in patients with borderline pathology, either as a temporary deterioration of their clinical state during hospitalization or as resistance to leaving the hospital after a few weeks of care. It is the team's responsibility to analyze the relational dynamics and psychic modifications involved in the therapeutic relationship, considering the patient's past experiences and their implications for meeting and separation. With proper attention, the discharge process can become a represented and surmountable separation.

Finally, the transferential aspects within the institution cannot be understood without considering the transference-countertransference dynamics between patients and the institution itself. This work occurs during weekly caregiver-client meetings involving all patients and the care team. In line with the tradition of institutional psychotherapy, individuals can only be effectively treated if the institution itself is also treated (13, 18, 20, 65). These therapeutic moments provide an opportunity to question the framework and purpose of care, as well as the patients' and the group's appropriation of them. They also serve as a therapeutic moment for the institution, allowing for ongoing reflection. The agenda of these meetings is determined in real-time, based on the group's and caregivers' current issues. Improvisation creates space for the emergence of affects and primary processes, which are the foundation of collective work. With borderline symptoms, the psychic space spills over into reality (66, 67), and the patient group becomes a privileged context for the projection movements of each patient. These moments enable the gathering and containment of projective identification, persecution, and disorganization arising from daily events in the unit. Moreover, the use of peer identification is a powerful therapeutic process in adolescence. Treatment within the group setting provides an opportunity to develop reflexivity and mentalization (68, 69). It also facilitates the collective construction and clarification of treatment objectives, stages, and encountered difficulties. Collective elaboration and containment work occur during group sessions and in post-group discussions among caregivers.

In summary, this work at the interface between the patient and their relational environment aims to address the relational aspects of borderline symptoms and enable the patient to differentiate between projection and reality in their daily interactions with others. This process of differentiation allows for a re-anchoring of these patients in reality.

## Individual therapeutic containment

This work on different levels of containment is crucial for the patient to establish a sense of individuality within the complex system of care that evolves and adapts over time.

### A. (Re)discovering a differentiated identity

During hospitalization, individual medical interviews are conducted regularly (though not necessarily on a daily basis). These interviews involve a nurse caregiver who has a closer connection to the adolescent's daily emotional experiences compared to the doctor. The nurse caregiver shares mediation times, meal times, and informal moments with the patient, in addition to the interview sessions. The main objective of these interviews is to restore the connection between emotions and mental representations, addressing the limited capacity for mentalization often observed in patients with borderline pathology (70). Adolescents with borderline pathology frequently struggle to make sense of the emotions they experience and the sudden changes in their emotional states. During the interviews, concrete relational situations are analyzed to understand how daily events impact the patient's emotions. Borderline affective instability is characterized by an exaggerated reactivity to seemingly insignificant triggers. However, these patients often have a history of significant emotional disruptions from a young age, and their emotional turmoil during adolescence is often linked to situations that hold more significance than they initially appear. These situations may include the fear of separation, relationship difficulties, or the feeling that their needs are not being met. Patients tend to anticipate negative events and perceive the world as threatening, which can lead them to generalize their experiences and interpret even minor situations as highly significant (28). During the interview, a thorough analysis of these relational situations allows the patient's conscious experiences to be compared with their unexpressed emotional and bodily reactions. This process aims to construct new shared representations and promote a deeper understanding of the patient's crisis. It is crucial to look beyond environmental factors and identify the underlying psychic events that triggered the patient's crisis. While the patient's environment may contribute to potential risks, understanding how the patient experienced specific relational events provides valuable insights into the mechanisms of the crisis and helps outline the stages of psychological mobilization.

The individual interviews also serve to promote the subjectivation of the adolescent in the days following family interactions. During these interviews, adolescents have the opportunity to express their perceptions, understanding, and feelings regarding their parents' stories and emotions. This process helps enhance their perceptual abilities by incorporating the perspectives of the caregivers themselves. When these shared perceptions align, they are grounded in a common reality and reinforce the patient's confidence in their own perceptual capabilities. However, when perceptions differ, it opens up a discussion about relational reference points and potential causes underlying the emotions of family members, thus exploring the family history. This allows the patient to practice inferring their own and others' mental states and develop mentalizing abilities, which are key targets in mentalization-based therapies (69–72). Furthermore, the development of insight and reflexivity abilities, which involve self-reflection, is an important aspect of the patient's prognosis. By engaging in representative work on internal and interpersonal psychological processes, the patient can subjectively appropriate their experiences and consolidate their sense of self. The therapist tactfully points out paradoxes, contradictions, oppositions, and inconsistencies in the patient's discourse, allowing the adolescent to become aware of the internal conflicts at play and to develop independent thinking. The analysis of daily aspects in the here and now serves as a starting point for reflecting on past and recurring experiences. These therapeutic techniques share similarities with aspects of Transference-Focused Therapy, which focuses on therapeutic affective movements, and Dialectical Behavioral Therapy, which addresses Manichean functioning (63, 73, 74). During hospitalization, the level of support in the here and now is intensified for patients with more fragile selves. For patients with less severe borderline symptoms, the work conducted in these interviews enables them to acquire the psychological tools necessary to establish a new relationship with the world based on an understanding of the factors that contributed to the crisis and its emotional manifestations. However, for other patients, whose capacity to connect emotions and representations is too overwhelming, the therapeutic work will focus more intensely on reactivated past relational experiences in the therapeutic relationship with the caregivers during hospitalization. In this context, the meaning and explicitness of daily therapeutic interventions become crucial in facilitating this process of psychological connection.

Medical interviews provide a crucial space for discussing traumatic events, their causes, and their consequences, allowing patients to share their experiences with another human being. This opportunity for emotional sharing is supported by empathetic caregivers. Adolescents often experience isolated traumatic events, such as episodes of physical violence. However, the complex nature of trauma in borderline pathology makes it difficult for patients to recognize the abnormality of their experiences. Caregivers play a vital role in guiding patients regarding social norms (e.g., the abnormality and illegality of sexual intercourse between a 13-year-old and an adult) and addressing the emotional needs of young individuals as they develop (emotions, needs, constraints, desires, projects, etc.). Through the sharing of the patient's experiences, a sense of temporality is established within their narrative. This temporal framework is essential for addressing traumatic symptoms, as individuals often experience a loss of temporal landmarks. Distinguishing between the patient's individual story and the stories of their parents or family members also enables temporal and spatial differentiation. Through discussing life experiences with a compassionate listener, subjective appropriation of these experiences occurs, allowing for differentiation from the experiences of relatives. This psychological work contributes to reducing dissociative phenomena and establishing a stronger grounding in reality. The clinician assumes the role of a secondary environmental response, providing representation and validation that was lacking in the primary response. This identification, influenced by Ferenczi's concept of a primitive failure of the object's response to the subject's experiences, highlights the importance of later inscription of experiences that were not adequately processed. As Green (75) suggests, “the response through countertransference is the one that should have taken place on the part of the object”. Consequently, the psychological work surrounding these experiences helps reduce the risks of repetitive traumatic patterns and psychotic disintegration, aiming to restore the patient's relationship with reality (76). Crisis hospitalization also serves as a significant moment for the transformation of childhood images. The borderline crisis presents an opportunity to confront the images constructed since childhood with the reality of the parents as they are during treatment. It allows the adolescent to engage in a seesaw process, as described by Kestemberg (77), involving a transition from disappointment to self-conquest.

### B. Body therapies

After the initial phase of hospitalization, which may be characterized by a “honeymoon” period, patients often enter into conflictual therapeutic relationships with their caregivers, feeling that they are not receiving sufficient help in managing their symptoms. Additionally, due to decreased mentalization and insight capacities in patients with borderline pathology, their symptoms tend to manifest behaviorally and bodily, with self-aggressive behaviors, such as self-injury, being common. These self-injurious behaviors serve as visible expressions of underlying instability, which the patients themselves often struggle to identify. This presents another opportunity for shared representation work, which is conducted on a daily basis with the nurses and discussed in meetings with the doctors. Nurses propose hypotheses and draw connections between internal emotional experiences and events the patients have encountered during the day or week. To aid in this process, nurses utilize various tools to help patients identify and regulate their emotions. One such tool is the establishment of a written emotional report, which assists patients in labeling the fluctuations of their physical sensations and emotions throughout the week.

In the development of patients with borderline pathology, attention may shift toward both parents and one of them as a means of self-protection and/or protecting others. Early experiences of concern for a parent's compromised health, fear of separation, or other sources of worry can lead to heightened observational abilities directed toward the environment, while self-observation capacities may be diminished. Green (78) described how these early modalities, through the paradigm of the dead mother complex, affect the child's personality, resulting in difficulties perceiving and interpreting affects within oneself and the development of alexithymia. Bowlby (60) also proposed that early experiences of relational withdrawal from attachment figures have an impact on development, a finding supported by several studies linking insecure attachment and borderline symptoms (79–81). Adverse experiences can lead to distinct perceptual modifications, with relational withdrawal experiences correlating with hypersensitivity in facial emotion recognition and intrusion experiences associated with decreased emotional perceptual accuracy (82). In all cases, attention to the internal world is diminished, likely due to difficulties accessing early parental mirroring functions (83). During hospital treatment, the emotional regulation work conducted with caregivers enables patients to shift their attention to their internal world. Caregivers provide them with tools to apprehend and regulate their emotions once they are identified and understood. While putting emotions into words is an important tool, it is not the only one. Breathing exercises, such as abdominal breathing, cardiac coherence exercises, and mindfulness, are fundamental. Caregivers, nurses, or body therapists may also offer massages or other body treatments that promote a sense of calm. In extreme cases, patients may find comfort in being wrapped in blankets or sheets, lying in a hammock, or resting on cushions in a soothing environment. Body care is also provided through various activities, such as sports, yoga, dance therapy, relaxation exercises, circus arts, or socio-aesthetic care, offered on a daily basis.

Caregivers and therapists who organize these activities serve as conduits for accessing affective and bodily integration, aiming to construct psychic representations from threatening, absent, or dissociated bodily perceptions. This “space outside-the-self” can only be accessible to the psyche if it can be represented, and pre-representations registered in the body can only be expressed through interaction (84). The experience of affect arises through the shaping of a relational schema. The development of a sense of self, which is lacking in patients, emerges from interactions with others and involves both psychological relationships (gaining meaning, sharing representations) and bodily aspects (tonic support, affective holding, handling). Similar to how an infant relies on the reflexive ability of their early environment, adolescents depend on the capacity of their environment to engage relationally, affectively, and bodily to support their tonicity (85). The internalization of bodily and relational elements, connected through processes of symbolization, allows adolescents to form an envelope that contains psychic, affective, and sensory contents, similar to Anzieu's (86) concept of the Skin-Ego. Just as infants, during the separation that inaugurates mother-child differentiation, maintain their unity by symbolically linking perceptions and sensations to psychically survive the absence, adolescents also rely on this process to navigate separation without exhausting themselves.

In addition to body therapies, alleviating borderline symptoms may involve pharmacological treatments, although their effectiveness is somewhat limited according to randomized controlled trials (87, 88). Medications are primarily prescribed based on the identified clinical dimensions present in borderline pathology or its numerous comorbidities, such as anxiety, self-aggression, major mood variations, and insomnia. Second-generation antipsychotics with anxiolytic aims are often preferred for a limited duration of a few weeks or months (89). The choice of medication depends on the patient's psycho-corporal profile, considering the specificities of adolescent metabolism that may result in more side effects compared to adults receiving the same dosage, particularly metabolic syndrome (90, 91). The appropriate timing for medication prescription is also considered, with the need to establish a therapeutic alliance and address any resistance or concerns regarding medication, as the fantasy of dependence can be particularly significant for patients and parents. Therefore, addressing this dimension in parallel is crucial to improve compliance. Additionally, the presence of various addictions often requires regular consultations with specialized addiction professionals during and/or after hospitalization.

### C. Autonomy toward the exit

In the final phase of care, the focus is on promoting autonomy as the adolescent prepares for discharge from the hospital. The patient gradually engages in out-of-hospital activities, initially accompanied by staff and eventually on their own in a safe environment near the hospital. This process allows them to re-establish autonomy and learn to manage stressful situations independently, while still having the option to seek support if needed. Weekend leaves also provide opportunities to practice autonomy and ensure the safety of both internal and external spaces. During this stage, it is important for the patient to distinguish between manageable and more challenging situations and symptoms, and to develop a safety plan that outlines coping strategies and available resources for each level of stress. This plan may include activities such as listening to music, reading, drawing, engaging in breathing exercises, going for walks with a trusted person, reaching out to caregivers or close individuals, practicing body calming exercises, taking prescribed medications, or contacting the referring psychiatrist or the hospital. As discharge approaches, the focus shifts to preparing the adolescent for greater autonomy outside of the hospital setting. This includes gradually reintegrating into daily life, spending time alone at home, engaging in social activities with friends, and taking trips to the city. It is essential to consider the stability of the patient's clinical state at discharge, as borderline symptoms may require multiple hospitalizations to achieve regulation. Therapeutic strategies can be employed to minimize the risk of suicidal acts, such as implementing a watch and call system or planning for sequential re-hospitalization if necessary.

If medication has been part of the treatment, this phase allows the adolescent's input to be considered when adjusting the treatment plan, as they will ultimately decide whether to continue taking medication on a daily basis after discharge. As the patient identifies their sources of difficulty, they take progressive steps toward moving forward and finding meaning and purpose in their internal states. Discharge signifies a planned and announced end to the hospitalization, representing a positive change in the therapeutic relationship, which is often different from the initial patterns experienced by hospitalized patients. It is also a time for parental guidance, managing attenuated symptoms that still carry a risk of recurrence. Parental guidance involves learning to manage risk while providing a containing function for the child.

At the end of hospitalization, in addition to the significant reduction in borderline symptoms, the therapist evaluates various factors, including the evolution of self-esteem, the level of hope for both the adolescent and their parents, the insight of each individual, and the ability to engage the family group in action. These objectives align with the aims of successful psychotherapy, which include building confidence, exploring conflicts, regulating emotions, and managing separations and reunions. However, the temporal dynamics of hospitalization differ significantly, and the emotional intensity experienced over the course of a few weeks plays a crucial role in shaping the psychological impact of these experiences.

In summary, the last stage of care focuses on the patient's own capacities and their ability to translate and regulate their internal states. The goal is to support them in resuming their life journey with increased serenity and equipped with the necessary skills and resources.

## Conclusion

The quote by Marcus Aurelius, “May I be given the strength to bear what cannot be changed and the courage to change what can be, but also the wisdom to distinguish one from the other,” encapsulates the therapeutic approach for adolescents with borderline symptoms.

In pursuit of the holistic objective, the three levels of containment, crucial in the treatment of adolescents with borderline symptoms, are interconnected and intertwined. They progress sequentially over time, with global containment being planned and organized prior to the patient's admission, followed by local containment during the hospitalization period, and individual containment becoming more prominent toward the end of the hospitalization. At each level, the preventive and therapeutic objectives are inherently linked to the analysis of the contributing factors. The hospital objectives align with those of the outpatient psychotherapeutic model for patients with borderline symptoms. These objectives include establishing a structured framework, involving the parents, actively engaging therapists with the adolescent, addressing resistance factors associated with the disorder, and restoring coherence and psychological continuity (92). For the most severe cases, additional layers of containment are implemented, requiring strong cohesion and collective creativity within the care teams. It would be beneficial in the future to compare this holistic model with singular models to quantitatively assess how different theoretical approaches complement each other within a comprehensive perspective. Overall, this model aligns with the current global health trend, which emphasizes transnational health issues, determinants, and solutions for enhancing overall wellbeing. By providing a detailed description of the individual's environment, it enables better preventive and therapeutic interventions to be implemented.

The therapeutic work aims to assist these patients in achieving a more balanced position within their environment. This involves fostering a sense of differentiation from others (psychic separation) while maintaining emotional connection in a secure manner. The goal is to support them in representing the adversities they have experienced and developing protective and coping strategies to navigate these experiences. It also involves reactivating the process of autonomy-dependence that is inherent to adolescence. Furthermore, the aim is to help the family identify their challenges, difficulties, and available individual and collective resources. Ultimately, the objective is to help the adolescent achieve inner harmony and find peace within their environment.

## Author contributions

Conceptualization: MR, RR, and MC. Methodology: MR, LB, KK, and JB. Clinical investigation: LB, JB, and MR. Original draft preparation: RR and MR. Reviewed by: MC, JB, LB, and KK. Supervision: MC. The manuscript was written by MR and approved by all co-authors. All authors contributed to the article and approved the submitted version.

## References

[1] Linehan MM. Understanding Borderline Personality Disorder: The Dialectic Approach Manual. New York, NY: Guilford Press. (1995)

[2] Steele H Siever L. An attachment perspective on borderline personality disorder: advances in gene-environment considerations. Curr Psychiatry Rep. (2010) 12:61–7. 10.1007/s11920-009-0091-0 20425312

[3] Sharp C Fonagy P. Practitioner Review: Borderline personality disorder in adolescence–recent conceptualization, intervention, and implications for clinical practice. J Child Psychol Psychiatry. (2015) 56:1266–88. 10.1111/jcpp.12449 26251037

[4] Gunderson JG Herpertz SC Skodol AE Torgersen S Zanarini MC. Borderline personality disorder. Nat Rev Dis Prim. (2018) 4:18029. 10.1038/nrdp.2018.29 29795363

[5] Distel MA Middeldorp CM Trull TJ Derom CA Willemsen G Boomsma DI. Life events and borderline personality features: the influence of gene-environment interaction and gene-environment correlation. Psychol Med. (2011) 41:849–60. 10.1017/S0033291710001297 20594379

[6] Stepp SD Keenan K Hipwell AE Krueger RF. The impact of childhood temperament on the development of borderline personality disorder symptoms over the course of adolescence. Borderline Person Dis Emot Dysreg. (2014) 1:18. 10.1186/2051-6673-1-18 26064524 PMC4459747

[7] Porter C Palmier-Claus J Branitsky A Mansell W Warwick H Varese F. Childhood adversity and borderline personality disorder: a meta-analysis. Acta Psychiatr Scand. (2020) 141:6–20. 10.1111/acps.13118 31630389

[8] Marcelli D Braconnier A Tandonnet L. Adolescence et Psychopathologie. Paris: Dunod. (2018).

[9] Becker DF Grilo CM Edell WS McGlashan TH. Diagnostic efficiency of borderline personality disorder criteria in hospitalised adolescents : comparison with hospitalised adults. Am J Psychiatry. (2002) 159:2042–7. 10.1176/appi.ajp.159.12.2042 12450954

[10] Kernberg OF. Borderline Conditions and Pathological Narcissism. New York, NY: Jason Aronson. (1975).

[11] Corcos M Lamas C Robin M. Borderline functioning in adolescence: psychopathology and psychodynamic clinic. Front Psychiatr. (2023).

[12] Ougrin D Latif S. Specific psychological treatment versus treatment as usual in adolescents with self-harm: systematic review and meta-analysis. Crisis. (2011) 32:74–80. 10.1027/0227-5910/a000060 21616756

[13] Delion P. Thérapeutiques institutionnelles. EMC-Psychiatr. (2001) 37:161–6.

[14] Robin M Cassini L Cornac X Pinardon M Polge C Pommeret S. La psychothérapie institutionnelle, terreau d'intelligence collective. L'Inf Psych. (2019) 95:653–60.

[15] Robin M Douniol M Pham-Scottez A Gicquel L Delvenne V Nezelof S. Specific pathways from adverse experiences to bpd in adolescence: a criteria-based approach of trauma. J Pers Disord. (2021) 35:94–110. 10.1521/pedi_2021_35_523 33999657

[16] Winnicott DW. Playing and Reality. 2nd Edn. London: Routlege (1971).

[17] Racamier PC. L'esprit des soins. Vinsobres: Editions du Collège. (2001).

[18] Oury J. Le Collectif: le Séminaire de Sainte-Anne. Nîmes: Champ Social Éditions. (1999).

[19] Roussillon R. Propositions pour une théorie des dispositifs thérapeutiques à mediations. Le Carnet Psy. (2010) 141:28–31. 10.3917/lcp.141.0028

[20] Tosquelles F. Éducation et Psychothérapie Institutionnelle. Paris: Hiatus. (1984).

[21] Racamier PC. Le Psychanalyste Sans Divan. Paris: Payot. (1993).

[22] Hochmann J. Soigner, éduquer, instituer, raconter. Histoire et actualité des traitements institutionnels des enfants psychiquement troublés. Revue Française Psych. (2006) 70:1043–63. 10.3917/rfp.704.1043

[23] Diehl K Jansen C Ishchanova K Hilger-Kolb J. Loneliness at universities: determinants of emotional and social loneliness among students. Int J Environ Res Public Health. (2018) 15:1865. 10.3390/ijerph15091865 30158447 PMC6163695

[24] Robin M Votadoro P. En France, en 2022, des enfants et adolescents meurent de souffrance psychique par manque de soins et de prise en compte sociétale. J Monde (2022).

[25] Stepp SD Whalen DJ Scott LN Zalewski M Loeber R Hipwell AE. Reciprocal effects of parenting and borderline personality disorder symptoms in adolescent girls. Dev Psychopathol. (2014) 26:361–78. 10.1017/S0954579413001041 24443951 PMC4103652

[26] Bourvis N Aouidad A Cabelguen C Cohen D Xavier J. How do stress exposure and stress regulation relate to borderline personality disorder? Front Psychol. (2017) 8:2054. 10.3389/fpsyg.2017.02054 29250007 PMC5714931

[27] Cicchetti D Handley ED. Methylation of the glucocorticoid receptor gene, nuclear receptor subfamily 3, group C, member 1 (NR3C1), in maltreated and nonmaltreated children: associations with behavioral undercontrol, emotional lability/negativity, and externalizing and internalizing symptoms. Dev Psychopathol. (2017) 29:1795–806. 10.1017/S0954579417001407 29162187 PMC5718163

[28] Robin M Belbèze J Pham-Scottez A Shadili G Peres V Silva J. Paradoxes in borderline emotional dysregulation in adolescence: influence of parenting, stressful life events, and attachment. Front Psychiatry. (2021) 12:735615. 10.3389/fpsyt.2021.735615 34744826 PMC8566741

[29] Lieb K Zanarini MC Schmahl C Linehan MM Bohus M. Borderline personality disorder. Lancet. (2004) 364:453–61. 10.1016/S0140-6736(04)16770-6 15288745

[30] Ibrahim J Cosgrave N Woolgar M. Childhood maltreatment and its link to borderline personality disorder features in children: a systematic review approach. Clin Child Psychol Psychiatry. (2018) 23:57–76. 10.1177/1359104517712778 28617046

[31] Milot T Collin-Vézina D Godbout N. Trauma Complexe: Comprendre, Évaluer et Intervenir. Québec: Presses de l'Université de Québec (2018).

[32] Lord S. Systemic work with clients with a diagnosis of Borderline Personality Disorder. J Fam Ther. (2007) 29:203–21. 10.1111/j.1467-6427.2007.00382.x

[33] Rosolato G. (1976). Le non-dit. Nouvelle Revue de psychanalyse n° 14 (Du secret).

[34] Abraham N Torok L. L'écorce et le Noyau. Paris: Flammarion (1978).

[35] Guilé JM Boissel L Alaux-Cantin S La Rivière SG. Borderline personality disorder in adolescents: prevalence, diagnosis, and treatment strategies. Adol Health Med Ther. (2018) 9:199–210. 10.2147/AHMT.S156565 30538595 PMC6257363

[36] Choi H. Family systemic approaches for borderline personality disorder in acute adult mental health care settings. J Family Ther. (2018) 39:155–73. 10.1002/anzf.130827409075

[37] Bergeret J. Les États-Limites. Ed Payot. (1984).

[38] Robin M. Ado désemparé Cherche Société Vivante. Paris: Ed Odile Jacob. (2017).

[39] Ilagan GS Choi-Kain LW. General psychiatric management for adolescents (GPM-A) with borderline personality disorder. Curr Opin Psychol. (2021) 37:1–6. 10.1016/j.copsyc.2020.05.006 32634737

[40] Rathus JH Miller AL. DBT Skills Manual for Adolescents. New York, NY: Guilford Press. (2015).

[41] Santisteban DA Muir JA Mena MP Mitrani VB. Integrative borderline adolescent family therapy: meeting the challenges of treating adolescents with borderline personality disorder. Psychotherapy. (2003) 40:251–64. 10.1037/0033-3204.40.4.251 25663719 PMC4317327

[42] Hoffman PD Fruzzetti AE Buteau E Neiditch ER Penney D Bruce ML. Family connections: a program for relatives of persons with borderline personality disorder. Fam Process. (2005) 44:217–25. 10.1111/j.1545-5300.2005.00055.x 16013747

[43] Rajalin M Wickholm-Pethrus L Hursti T Jokinen J. Dialectical behavior therapy-based skills training for family members of suicide attempters. Arch Suicide Res. (2009) 13:257–63. 10.1080/13811110903044401 19590999

[44] Pearce J Jovev M Hulbert C McKechnie B McCutcheon L Betts J. Evaluation of a psychoeducational group intervention for family and friends of youth with borderline personality disorder. Borderlin Person Dis Emot Dysreg. (2017) 4:5. 10.1186/s40479-017-0056-6 28352470 PMC5364611

[45] Guillén V Díaz-García A Mira A García-Palacios A Escrivá-Martínez T Baños R. Interventions for family members and carers of patients with borderline personality disorder: a systematic review. Fam Process. (2021) 60:134–44. 10.1111/famp.12537 32304101

[46] Mooren Tvan Ee E Hein I Bala J. Combatting intergenerational effects of psychotrauma with multifamily therapy. Front Psychiatry. (2023) 13:867305. 10.3389/fpsyt.2022.867305 36819942 PMC9929345

[47] Bernstein DP Cohen P Velez CN Schwab-Stone M Siever LJ Shin-Sato L. Prevalence and stability of the DSM-III-R personality disor- ders in a community-based survey of adolescents. Am J Psychiatry. (1993) 150:1237–43. 10.1176/ajp.150.8.1237 8328570

[48] Levy KN Becker DF Grilo CM Mattanah JJ Garnet KE Quinlan DM. Concurrent and predictive validity of the personality disorder diagnosis in adolescent inpatients. Am J Psychiatry. (1999) 156:1522–8. 10.1176/ajp.156.10.1522 10518161

[49] Miller AL Muehlenkamp JJ Jacobson CM. Fact or fiction: diagnosing borderline personality disorder in adolescents. Clin Psychol Rev. (2008) 28:969–81. 10.1016/j.cpr.2008.02.004 18358579

[50] Chabrol H Montovany A Chouicha K Callahan S Mullet E. Frequency of borderline personality disorder in a sample of French high school students. Can J Psychiatr Rev. (2001) 46:847–9. 10.1177/070674370104600909 11761637

[51] Johnson JG Cohen P Kasen S Skodol AE Oldham JM. Cumulative prevalence of personality disorders between adolescence and adulthood. Acta Psychiatr Scand. (2008) 118:410–3. 10.1111/j.1600-0447.2008.01231.x 18644003

[52] Zanarini MC Horwood J Wolke D Waylen A Fitzmaurice G Grant BF. Prevalence of DSM-IV borderline personality disorder in two community samples: 6,330 English 11-year-olds and 34,653 American adults. J Pers Disord. (2011) 25:607–19. 10.1521/pedi.2011.25.5.607 22023298 PMC4678770

[53] Zittel Conklin C Westen D. Borderline personality disorder in clinical practice. Am J Psychiatry. (2005) 162:867–75. 10.1176/appi.ajp.162.5.867 15863787

[54] Robin M. Etat Limite, Personnalité Borderline, ou Crise Borderline à L'adolescence? Troubles de la Personnalité Borderline à L'adolescence. Paris: Ed. Dunod (2013).

[55] Ainsworth M Witting B. Attachment and the exploratory behavior of one-year-olds in a strange situation. In BM Foss, Editor, Determinants of Infants Behavior. London: Methuen (1969).

[56] Ainsworth M Blehar M Waters E. Patterns of Attachment: A Psychological Study of the Strange Situation. Hillsdale: Erlbaum. (1978).

[57] 51 Guédeney N Guédeney A. L'attachement, Approche Théorique. Du Bébé à la Personne Âgée. Paris: Masson (2016).

[58] Main M Hesse E. Disorganized/disoriented infant behavior in the strange situation, lapses in the monitoring of rasoning and discourse during the parent's Adult Attachment Interview, and dissociative states. In M Ammaniti, D Stern, editors Attachment and Psycho-Analysis. Rome: Guis, Laterza, and Figl, 80–140 (1992).

[59] Bowlby J. Attachment and Loss, Vol. 1. New York, NY: Basic Books (1969).

[60] Bowlby J. A Secure Base: Parent-Child Attachment and Healthy Human Development. New York, NY: Basic Books. (1988).

[61] Robin M. L'hospitalisation en psychiatrie à l'adolescence : une situation étrange? L'observation de l'adolescent hospitalisé à la lumière de la théorie de l'attachement. Enfances Psy. (2019) 3:155–63. 10.3917/ep.083.0155

[62] Clarkin JF Yeomans FE Kernberg OF. Psychotherapy of Borderline Personality: Focusing on Object Relations. Washington, DC: American Psychiatric Publishing Inc (2006).

[63] Foelsch PA Odom AE Kernberg OF. Modifications à la psychothérapie focalisée sur le transfert (PFT) pour le traitement des adolescents avec une identité diffuse. Santé Mentale au Québec (2008) 33:37–60. 10.7202/018472ar 18795195

[64] Freud S. La Dynamique du Transfer, Dans La Technique Psychanalytique. Paris: PUF (2010).

[65] Ducarre C. Comment contenir ce qui nous est étranger: Les réunions soignants-soignés à Orgemont. Cliniques. (2011) 1:78–92. 10.3917/clini.001.0078

[66] Jeammet P. Réalité externe et réalité interne, importance et spécificité de leur articulation à l'adolescence. Rev Française Psychanalyse (1980) 4:481–521.

[67] Azoulay J. Les Psychothérapies Institutionnelles, Psychanalyse et Psychothérapie. Paris: Flammarion (1996).

[68] Bion WR. Une théorie de l'activité de pensée (A theory of Thinking), in Réflexion faite. PUF. (1983) 1983:125–35.

[69] Bo S Vilmar JW Jensen SL Jørgensen MS Kongerslev M Lind M. What works for adolescents with borderline personality disorder: towards a developmentally informed understanding and structured treatment model. Curr Opinion Psychol. (2021) 37:7–12. 10.1016/j.copsyc.2020.06.008 32652486

[70] Fonagy P Bateman A. The development of borderline personality disorder–a mentalizing model. J Pers Disord. (2008) 22:4–21. 10.1521/pedi.2008.22.1.4 18312120

[71] Bo S Sharp C Beck E Pedersen J Gondan M Simonsen E. First empirical evaluation of outcomes for mentalization-based group therapy for adolescents with BPD. Personal Disord. (2017) 8:396–401. 10.1037/per0000210 27845526

[72] Quek J Melvin GA Bennett C Gordon MS Saeedi N Newman LK. Mentalization in adolescents with borderline personality disorder: a comparison with healthy controls. J Pers Disord. (2019) 33:145–63. 10.1521/pedi_2018_32_336 29469664

[73] Linehan MM. Skills Training Manual for Treating Borderline Personality Disorder. New York, NY: Guilford Press. (1993).

[74] Klein DA Miller AL. Dialectical behavior therapy for suicidal adolescents with borderline personality disorder. Child Adolesc Psychiatr Clin N Am. (2011) 20:205–16. 10.1016/j.chc.2011.01.001 21440851

[75] Green A. La Folie Privée. Paris: Gallimard. (1990).

[76] Robin M Essadek A Corcos M Shadili G. High-risk sexual behaviours, from the neurotica to complex trauma: psychopathologies of repetition. Int J Psychoanal. (2021) 102:906–31. 10.1080/00207578.2021.1939036 34396899

[77] Kestemberg E. ≪ La crise de l'adolescence. Rev Fr Psychanal. (1980) 44:3–4.

[78] Green A. La Mère Morte, in Narcissisme de vie, Narcissisme de Mort. Paris: Éditions de Minuit, 222–253 (1980).

[79] Schuder MR Lyons-Ruth K. “Hidden trauma” in infancy: attachment, fearful arousal, and early dysfunction of the stress response system. In:Osofsky JD, editor Young Children and Trauma: Intervention and Treatment. New York, NY: Guilford Press, (2004), 69–104.

[80] Lyons-Ruthn K Bureau JF Easterbrooks MA Obsuth I Hennighausen K Vulliez-Coady L. Parsing the construct of maternal insensitivity: distinct longitudinal pathways associated with early maternal withdrawal. Attach Hum Dev. (2013) 15:562–82. 10.1080/14616734.2013.841051 24299135 PMC3861901

[81] Briere J Runtz M Eadie E Bigras N Godbout N. Disengaged parenting: Structural equation modeling with child abuse, insecure attachment, and adult symptomatology. Child Abuse Neglect. (2017) 67:260–70. 10.1016/j.chiabu.2017.02.036 28284895

[82] Robin M Belbèze J Pham-Scottez A Speranza M Shadili G Silva J. Adversity, attachment and emotion recognition in BPD adolescents: the distinct roles of disengaged and controlling environment. BMC psychology. (2022) 10:89. 10.1186/s40359-022-00788-7 35379363 PMC8981788

[83] Fonagy P Luyten PA. Developmental, mentalization-based approach to the understanding and treatment of borderline personality disorder. Dev Psychopathol. (2009) 21:1355–81. 10.1017/S0954579409990198 19825272

[84] Aulagnier P. La Violence de L'interprétation. 7th Edn. Paris: PUF.

[85] Corcos M. Le silence des émotions. Clinique Psychanalytique des états Vides D'affects. Paris : Dunod (2011).

[86] Anzieu D. Le Moi–Peau. Paris: Editions Dunod (1985).

[87] Biskin RS. Treatment of borderline personality disorder in youth. J Can Acad Child Adol Psychiatr. (2013) 22:230–4. 23970912 PMC3749897

[88] Stoffers JM Lieb K. Pharmacotherapy for borderline personality disorder–current evidence and recent trends. Curr Psychiatry Rep. (2015) 17:534. 10.1007/s11920-014-0534-0 25413640

[89] Chanen AM Thompson KN. Prescribing and borderline personality disorder. Austr Prescriber. (2016) 39:49–53. 10.18773/austprescr.2016.019 27340322 PMC4917638

[90] Correll CU Carlson HE. Endocrine and metabolic adverse effects of psychotropic medications in children and adolescents. J Am Acad Child Adolesc Psychiatry. (2006) 45:771–91. 10.1097/01.chi.0000220851.94392.30 16832314

[91] Garcia G Logan GE Gonzalez-Heydrich J. Management of psychotropic medication side effects in children and adolescents. Child Adolesc Psychiatr Clin N Am. (2012) 21:713–38. 10.1016/j.chc.2012.07.012 23040898

[92] Weiner AS Ensink K Normandin L. Psychotherapy for borderline personality disorder in adolescents. Psychiatr Clin North Am. (2018) 41:729–46. 10.1016/j.psc.2018.07.005 30447735

